# Reduction of HIV-1 Reservoir Size and Diversity After 1 Year of cART Among Brazilian Individuals Starting Treatment During Early Stages of Acute Infection

**DOI:** 10.3389/fmicb.2019.00145

**Published:** 2019-02-11

**Authors:** Thaysse Ferreira Leite, Edson Delatorre, Fernanda Heloise Côrtes, Ana Cristina Garcia Ferreira, Sandra Wagner Cardoso, Beatriz Grinsztejn, Michelle Morata de Andrade, Valdilea Gonçalves Veloso, Mariza Gonçalves Morgado, Monick Lindenmeyer Guimarães

**Affiliations:** ^1^Laboratório de AIDS e Imunologia Molecular, Instituto Oswaldo Cruz, Fundação Oswaldo Cruz, Rio de Janeiro, Brazil; ^2^Laboratório de Pesquisa Clínica em DST e AIDS, Instituto Nacional de Infectologia Evandro Chagas, Fundação Oswaldo Cruz, Rio de Janeiro, Brazil

**Keywords:** HIV-1, reservoir, diversity, early cART, acute infection

## Abstract

The aim of early combined antiretroviral therapy (cART) of HIV is to limit the seeding of the viral reservoir during the initial phase of infection and, consequently, decrease intrahost viral diversity. Here, we assessed the effect of early cART on size and complexity of the proviral reservoir. Peripheral blood mononuclear cell (PBMC) and plasma samples were obtained from ten HIV-infected Brazilian individuals diagnosed at the acute phase of infection, before (PRE_ART_) and 12 months (M12_ART_) after suppressive cART. HIV proviral reservoir size was determined by quantitative real-time PCR; intrahost viral diversity of the *env* C2-V3 region was assessed by single genome amplification or next-generation sequencing in PBMC and plasma, respectively. Mean nucleotide diversity (π) and normalized Shannon entropy (H_SN_) were used to infer the complexity of the viral population. Compared to PRE_ART_, M12_ART_ saw an immunological recovery with a gain of ∼200 CD4^+^ T cells (*P* = 0.008) and a normalization of the CD4/CD8 ratio [1.0 (IQR: 0.88–1.18), *P* = 0.016], as well as a significant decrease in HIV-1 RNA (∼4 log, *P* = 0.004) and DNA (∼1 log, *P* = 0.002) levels. The median time to achieve viral suppression was 3 months (IQR: 2.8–5.8 months). The high intermixing between sequences from both visits suggests that the HIV-1 DNA reservoir remained remarkably stable under cART. After 1 year of cART, there was a minor reduction in proviral π (Pre_ART_ = 0.20 vs. M12_ART_ = 0.10; *P* = 0.156) but a significant decrease in H_SN_ (Pre_ART_ = 0.41 vs. M12_ART_ = 0.25; *P* = 0.019). We found no correlation between π or H_SN_ at Pre_ART_ and the rate of HIV DNA decay, T CD4^+^ counts, or CD4/CD8 ratio at M12_ART_. Based on a small cohort of Brazilian infected individuals under early cART and analyses of the *env* region, 1 year of follow-up suggested a reservoir size reduction, allowed a significant decrease of HIV-1 complexity, and achieved immunological restoration regardless of the initial HIV-1 plasma viral load, CD4^+^ T cell counts, or HIV-1 subtype. However, further studies in the Brazilian setting aiming a longer follow-up and larger cohort are required in this field.

## Introduction

Combined antiretroviral therapy (cART) suppresses HIV-1 replication and reduces morbidity and mortality, but does not eradicate HIV-1 infection, as a low but persistent level of HIV-1 can still be detected in plasma and cell reservoirs (Chun et al., 1997; Kiselinova et al., 2015; Ghosn et al., 2018). Latent infected resting memory CD4^+^ T lymphocytes are the best known HIV-1 reservoir, which is established already during early infection. This reservoir includes cells with an integrated copy of the HIV-1 genome that is not expressed while the cells remain in a resting state (Chun et al., 2002; Douek et al., 2002) and is maintained mainly by the cells’ clonal expansion (Chomont et al., 2009, 2011; von Stockenstrom et al., 2015). However, it has been suggested that persistent virus replication may be an important contributor to its maintenance (Buzón et al., 2010; Yukl et al., 2010), particularly in lymphoid tissue sanctuary sites (Lorenzo-Redondo et al., 2016). Elimination/reduction of this latent reservoir represents a great hope for curing HIV-1 infection. As the reservoir is established during the acute phase of infection, early cART has been proposed as a means to restrict reservoir size and genetic complexity (Lori et al., 1999; Strain et al., 2005; Chomont et al., 2009; Josefsson et al., 2013).

HIV-1 sequence diversity is limited by the “genetic bottleneck effect” during sexual transmission, which selects viruses with the highest overall fitness (Carlson et al., 2014) but results in a more homogeneous viral population. However, in cohorts of men who have sex with men (MSM), the selection for fitter variants appears less stringent, resulting in infection being established by multiple founding viruses (Gottlieb et al., 2008; Li et al., 2010). Accordingly, the complexity of sequence diversity during early HIV-1 infection may affect the efficacy of cART in decreasing the reservoir, mainly due to immune and therapeutic escape mutations. Another potential escape route is through viral recombinants (Abrahams et al., 2009; Kearney et al., 2009; Bar et al., 2010; Li et al., 2010; Batorsky et al., 2011; Novitsky et al., 2011). Therefore, investigating the diversity of viral variants during primary infection may help evaluate viral evolution and predict clinical outcomes.

Residual replication has been proposed as a mechanism that maintains the HIV-1 reservoir during cART. Failure to block the viral replication cycle, enables renewed cellular infections and continuous replenishment of the HIV-1 DNA reservoir (Josefsson et al., 2013). Individuals who initiated cART during early infection, may present low viral diversity in peripheral blood mononuclear cell (PBMC) and plasma reservoirs after years on suppressive therapy, and the slight sign of viral replication indicates that the reservoir was maintained by homeostatic cell proliferation (Josefsson et al., 2013). A recent study demonstrated a faster decline in HIV DNA levels in early cART-treated patients with homogeneous viral populations after 6 months of therapy (Wang et al., 2017).

Understanding the dynamics of long-lived cellular HIV-1 reservoirs in individuals treated during primary infection, can direct the choice of long-term treatment regimens to achieve post-treatment control of HIV-1 in Brazil. Here, we investigated the effect of early cART during the initial Fiebig stages of HIV-1 acute infection on the size, diversity, and complexity of HIV-1 total DNA in the PBMC reservoir by examining HIV-1 populations prior and 1 year after cART initiation.

## Materials and Methods

### Subject Characteristics

A cohort of Brazilian individuals presenting recent HIV-1 infection has been followed-up since August 2013 at the Instituto Nacional de Infectologia Evandro Chagas (INI-FIOCRUZ). These individuals initiated cART immediately after diagnosis and have been described elsewhere (Ferreira et al., 2017). For the present study, we selected only individuals, who started cART during the acute phase (Fiebig II–V) (Fiebig et al., 2003) of HIV-1 infection (*n* = 10). Participants were recruited between December 2014 and October 2015, and had at least 1 year of successful cART after that. PBMC and plasma samples were obtained at the baseline visit (PRE_ART_) and 12 months after cART onset (M12_ART_), and were stored until use. The processing of all HIV samples was performed in accordance with institutional standard biosecurity and safety procedures at biosafety level 2. The study was approved by the INI Ethical Review Board (approval number 36859614.8.0000.5262), and all subjects gave written informed consent in accordance with the Declaration of Helsinki.

### CD4^+^ and CD8^+^ T Cell Counts and HIV-1 RNA Quantification

Peripheral blood CD4^+^ and CD8^+^ T cell counts were determined by flow cytometry using the MultiTest TruCount-Kit and MultiSet software on a FACSCalibur flow cytometer (BD Biosciences, United States). HIV-1 RNA in plasma was measured by the Abbot Real-Time HIV-1 Assay, whose lower limit of detection was 40 copies/mL (Abbott Laboratories, Germany).

### HIV-1 Total DNA Measurement in PBMCs

Total cellular DNA was extracted from cryopreserved PBMCs (1 × 10^7^ cells) obtained at PRE_ART_ and M12_ART_ using the QIAamp DNA Mini Kit (Qiagen, Germany). Cell-associated HIV-1 DNA was quantified using the Generic HIV^®^ DNA Cell Kit (Biocentric, France), following the manufacturer’s instructions. The assay’s lower limit of detection was 40 HIV DNA copies/10^6^ cells.

### HIV-1 DNA Single Genome Amplification (SGA)

Proviral DNA was extracted from PBMCs using the QIAamp DNA Blood Mini Kit (Qiagen, United States) according to the manufacturer’s instructions. HIV-1 quasispecies was obtained by SGA of a 552-bp fragment from the C2-V3 *env* region through nested PCR using Platinum Taq DNA polymerase (Invitrogen, United States) as described elsewhere (Delwart et al., 1993). Considering a Poisson distribution, at a dilution in which approximately 30% of amplicons are positive, a single amplifiable molecule is present about 80% of the time (Palmer et al., 2005). The PCR products were purified using the Illustra GFX PCR DNA and Gel Band Purification Kit (GE Healthcare, United Kingdom). Sequences were obtained using the ABI BigDye Terminator v.3.1 Cycle Sequencing Ready Reaction Kit (Applied Biosystems, United States) on an ABI 3130 Genetic Analyzer (Applied Biosystems). Sequences were assembled and edited using SeqMan 7.0 software (DNASTAR Inc., United States). APOBEC3G/F-mediated hypermutations were revealed by Hypermut software (Rose and Korber, 2000) and sequences showing ambiguous bases were excluded.

### HIV-1 *env* RNA Haplotypes Reconstruction From NGS Data

Viral RNA from plasma samples collected at PRE_ART_ (baseline) was extracted using the QIAamp Viral RNA Mini Kit (Qiagen, Germany). The cDNA was obtained by reverse-transcribed PCR using the SuperScript^TM^ III Reverse Transcriptase (Invitrogen, United States) and was then subjected to nested PCR for amplification of the *env* gene as described above. The resulting amplicons were made into a library using the Nextera^®^ XT DNA Library Prep Kit with unique barcodes from the Nextera^®^ XT Index Kit (Illumina, United States), following the manufacturer’s instructions. DNA sequencing was performed on a MiSeq instrument using MiSeq Reagent Nano Kit, v2 (500 cycles; Illumina, United States). Demultiplexed reads were trimmed to remove adaptors, low-quality bases (Q <25), and short reads (<100 bp), and then mapped against single-genome amplification consensus sequences from each patient using Geneious software v.9.1.8 (Kearse et al., 2012) with high mapping quality (MAQ ≥30). Alignment regions with at least 500× coverage were used for haplotype reconstruction with QuasiRecomb 1.2 (Marz et al., 2014), employing the flag “-conservative” to increase specificity. Only haplotypes with frequencies ≥1% were used for further analysis.

### HIV-1 Subtyping

Sequences were aligned with HIV-1 reference sequences from the Los Alamos database^1^ using ClustalW in MEGA 6 and were manually edited. The final *env* alignment covered positions 6,840–7,372 relative to the HXB2 genome. Maximum-likelihood (ML) phylogenetic trees were reconstructed with PhyML 3.0 (Guindon et al., 2010) using the most appropriate nucleotide substitution model selected with jModeltest v. 3.7 (Darriba et al., 2012). The approximate likelihood-ratio test (aLRT) was used to estimate the confidence of branching on the tree.

### Analyses of Viral Diversity

Complexity of the intrahost viral population was assessed through two diversity measures. The mean nucleotide diversity (π), an abundance-based functional index representing the average number of nucleotide differences between any two representatives of the population, was calculated in MEGA7 (Kumar et al., 2016). The normalized Shannon entropy (H_SN_), an abundance-based index that measures viral population diversity based on haplotype frequencies, was calculated in the R package vegan (Oksanen et al., 2017), after sample rarefaction to correct for bias in sample size (Gregori et al., 2016).

### Phenotypic Prediction of Co-receptor Usage

The quasispecies viral tropism was predicted based on the V3 amino acid sequence through the Geno2pheno algorithm (available at http://coreceptor.geno2pheno.org). The false positive rate cut-off was 10% for DNA, whereas RNA sequences were classified as CCR5 or non-CCR5-using viruses as described previously (Lengauer et al., 2007; Hayashida et al., 2017).

### Statistical Analyses

All statistical analyses were performed in GraphPad Prism v6 (GraphPad Software, United States). Variables between groups (unpaired) or from the same group from different visits (paired) were compared using the Mann–Whitney *U*-test or Wilcoxon test, respectively. Association between variables was evaluated using the Spearman’s rank correlation. Linear regression was used to calculate the rate of HIV-1 DNA variation between visits in each subject. *P*-values <0.05 were considered statistically significant.

### Sequence Availability

SGA *env* sequences have been submitted to GenBank under accession numbers MH765045–MH765329. NGS data have been deposited to the NCBI BioProject database under accession number PRJNA487221.

## Results

### Clinical, Epidemiological, and Immunological Characteristics of Early-Treated Individuals

The study included ten HIV-1-infected Brazilian individuals diagnosed during the acute phase of infection, with a median age at diagnosis of 28 years [interquartile range (IQR): 26–42 years]. All participants were MSM and started cART immediately after HIV-1 diagnosis. The cART regimen varied among participants, however, all included two co-formulated nucleoside reverse transcriptase inhibitors (lamivudine plus tenofovir) in combination with a non-nucleoside reverse transcriptase inhibitor (efavirenz, *n* = 5), a protease inhibitor (fosamprenavir/atazanavir, *n* = 2 each), or an integrase inhibitor (raltegravir, *n* = 1).

**FIGURE 1:**
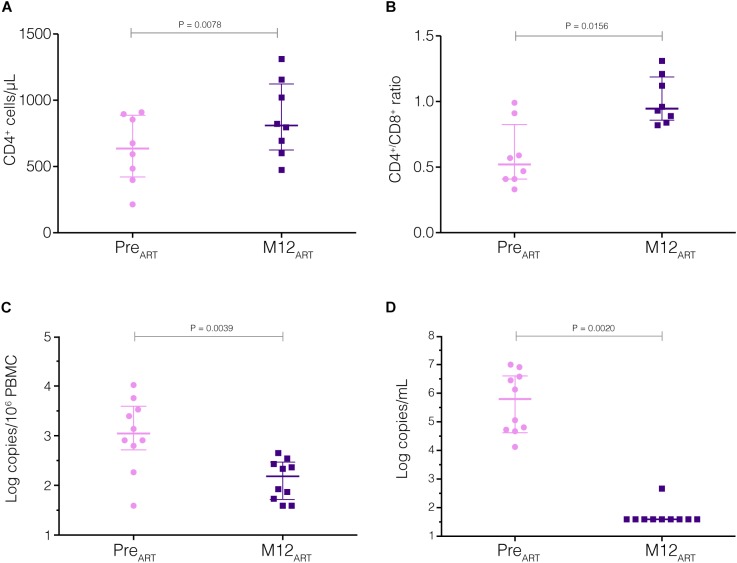
Immunological and virological measurements before and after cART initiation. CD4^+^ T cell counts **(A)**, CD4^+^/CD8^+^ ratios **(B)**, HIV-1 proviral load in PBMCs **(C)**, and HIV-1 viral load in plasma **(D)** were measured at Pre_ART_ and M12_ART_ visits (pink circles and blue squares, respectively). *P*-values <0.05 were considered statistically significant.

**FIGURE 2:**
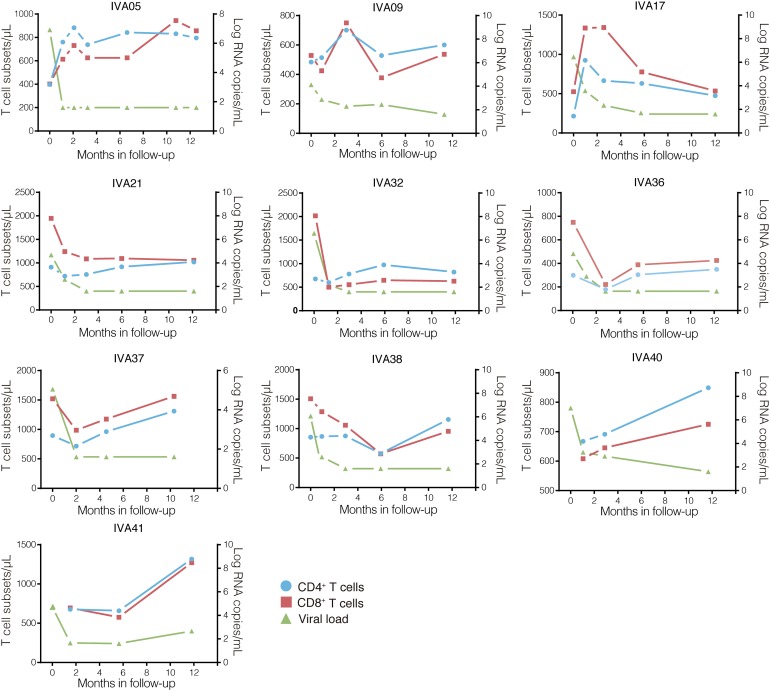
Clinical follow-up of the ten early-treated individuals. T cell counts (cells/μL; CD4^+^, blue circles; CD8^+^, red squares) and plasma RNA viral loads (log copies/mL, green triangles) values over time after cART onset (months) are shown on the left and right *Y*-axis, respectively. The individuals’ identifications are indicated at the top of each graph.

Prior to cART initiation (Pre_ART_ visit), median CD4^+^ and CD8^+^ T cell counts of all individuals were 634 and 1473 cells/mm^3^ (IQR: 420–886 for CD4^+^ and 526–1840 for CD8^+^ cells), respectively, and the CD4/CD8 ratio was 0.52 (IQR: 0.42–0.83). After 12 months on cART (M12_ART_), we observed a significant immunological recovery, with median CD4^+^ counts of 836 cells/mm^3^ (IQR: 719–1122) (Figure 1A, 2). This meant an average increase of ∼200 CD4^+^ T cells (*P* = 0.008) and a significant rise in the CD4/CD8 ratio [median of 1.0 (IQR: 0.88–1.18), *P* = 0.016] (Figure 1B, 2).

The immunological improvement observed 12 months after cART initiation was accompanied by a significant decrease in the median levels of HIV-1 total DNA in PBMCs and HIV-1 RNA in plasma (Figure 1C,D). The plasma HIV-1 viral load decreased drastically (∼4 log) between PRE_ART_ and M12_ART_ visits (from 5.86 to <1.6 log copies/mL, *P* = 0.004), whereas HIV-1 DNA in the PBMC compartment displayed a modest yet significant reduction (∼1 log, from 3.04 to 2.18 log copies/10^6^ PBMC, *P* = 0.002). The median time to achieve viral suppression (<1.6 log HIV RNA copies/mL) after the onset of cART was approximately 3 months (IQR: 2.8–5.8 months), and only one individual (IVA41) exhibited a minor viral rebound (2.66 log HIV RNA copies/mL) within the 12 months of follow-up (Figure 2). It should be noted, however, that sustained viral loads were recovered after that (data not shown).

### Sequence Analysis

To understand the effect of early cART initiation on HIV-reservoir diversity and complexity, we analyzed the intrahost viral population by SGA of the HIV-1 *env* gene before and after cART. Similar quantities of SGA HIV-1 proviral sequences were obtained from both visits’ samples: 15 (IQR: 15–16) in Pre_ART_ and 14.5 (IQR: 11–16) in M12_ART_ (*P* = 0.14). Additionally, we used NGS to evaluate HIV diversity in the individuals’ plasma samples at the Pre_ART_ visit. The median coverage per sample was 8,182 (IQR: 4,755–10,904) reads per base (Supplementary Figure S1), and after assembly between one to eight HIV-1 *env* haplotypes were reconstructed per sample.

**FIGURE 3:**
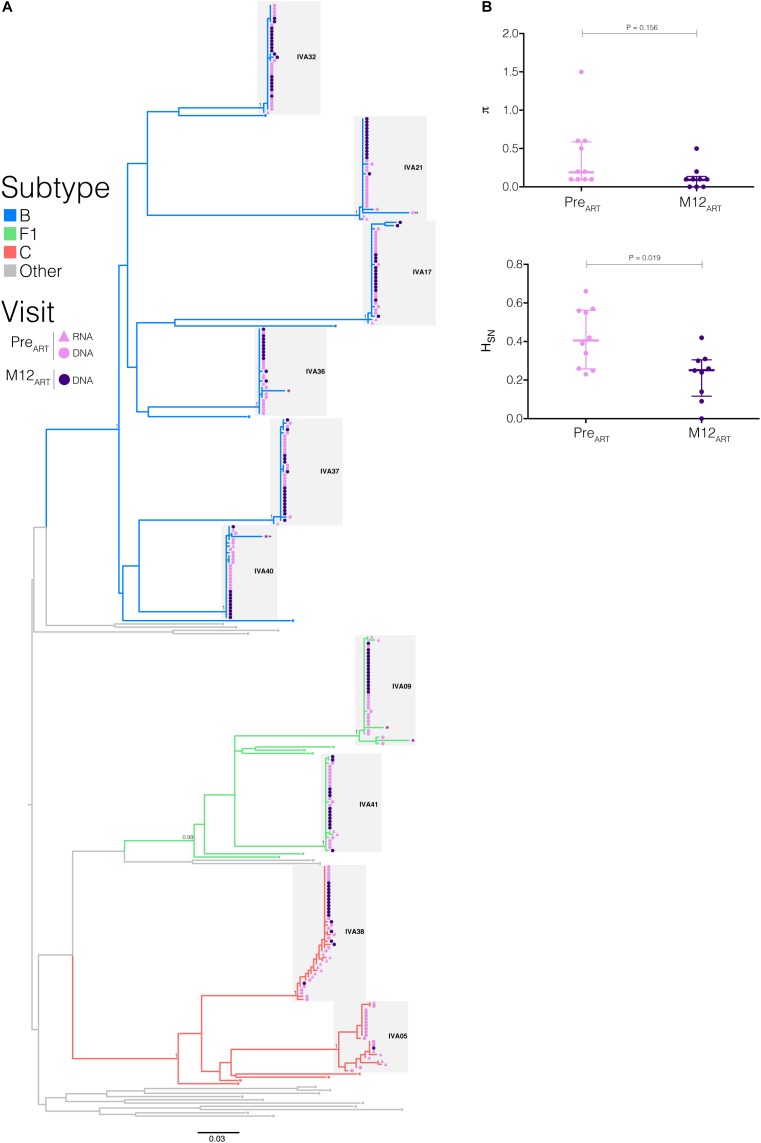
Impact of early cART initiation on intrahost HIV-1 population diversity. **(A)** ML phylogenetic tree of the *env* sequences from Pre_ART_ and M12_ART_ visits. Tips’ shapes represent the viral compartment (proviral DNA, circles; plasma RNA, triangles) and are color-coded according to the visit (Pre_ART_, pink; M12_ART_, purple). The branches’ colors agree with the subtype assignment as indicated in the legend. Clusters from each individual are indicated by shaded gray boxes. Branch supports (aLRT-SH) are indicated at key nodes. Tips shapes marked with an “H” indicate the presence of APOBEC3G-mediated G to A hypermutations and X4 labels highlight X4-tropic sequences. Horizontal branch lengths are proportional to the bar at the bottom indicating nucleotide substitutions per site. **(B)** Mean nucleotide diversity (π) and normalized Shannon entropy (H_SN_) indices were calculated from the proviral *env* sequences obtained at Pre_ART_ and M12_ART_ visits. Thick and thin lines represent the median and interquartile ranges, respectively. *P*-values <0.05 were considered statistically significant.

All sequences obtained from proviral DNA and plasma RNA branched together in highly supported monophyletic clusters (aLRT = 1) by subject (Figure 3A); suggesting that in all subjects infection resulted from a single or limited number of closely related viral variants. It is noteworthy that in four subjects (IVA32, IVA21, IVA17, and IVA37), the HIV-1 RNA sequences were the closest to the common ancestor of the subjects’ HIV-1-infecting lineage and outside the main cluster comprising the proviral variants. In the remaining subjects, the HIV-1 RNA sequences were intermixed with proviral DNA sequences. HIV-1 subtype B was the most frequent variant detected (*n* = 6), followed by subtypes F1 and C (*n* = 2 in each). R5-tropic viruses dominated the intrahost HIV-1 population in plasma and PBMCs at both visits, with only two individuals (IVA21 and IVA40) presenting a low frequency (6%) of X4-tropic viral clones in PBMCs at Pre_ART_ (Table 1 and Figure 3A). APOBEC3G/F-induced hypermutations were identified at Pre_ART_ in proviral sequences from only three individuals (IVA09, IVA36, and IVA40), with frequencies of 6–12%.

**Table 1:** Virological characteristics of HIV-1 early-infected individuals.

Patient ID	Fiebig stage	Subtype	Visit^1^	PMBC						Plasma							
				Log_10_	HIV DNA^2^	*env* clones	π (%)	H_SN_	Unique sequences (%)	R5 tropic (%)	Log_10_	HIV RNA^3^	*env* haplotypes	π (%)	H_SN_	R5 tropic (%)
IVA05	II	C	Pre_ART_	<1.6	14	1.6	0.6	46.6	100	6.9	7	2.7	0.2	100
			M12_ART_	<1.6	1	–	0.0	100	100	<1.6	–	–	–	–
IVA09	V	F1	Pre_ART_	2.8	17	0.5	0.4	26.6	100	4.1	2	1.0	0.0	100
			M12_ART_	1.7	15	0	0.0	6.7	100	<1.6	–	–	–	–
IVA17	V	B	Pre_ART_	4.0	15	0.1	0.3	26.6	100	6.5	2	0.3	0.1	100
			M12_ART_	2.5	15	0.5	0.2	26.6	100	<1.6	–	–	–	–
IVA21	V	B	Pre_ART_	2.9	16	0.6	0.2	25	93.7	4.7	2	0.3	0.1	100
			M12_ART_	1.9	14	0	0.1	14.3	100	<1.6	–	–	–	–
IVA32	V	B	Pre_ART_	3.4	15	0.1	0.3	20	100	6.6	1	–	0.0	100
			M12_ART_	2.4	18	0.1	0.3	22.2	100	<1.6	–	–	–	–
IVA36	IV	B	Pre_ART_	2.9	15	0.1	0.4	21.4	100	4.8	1	–	0.0	100
			M12_ART_	1.9	11	0.1	0.3	27.3	100	<1.6	–	–	–	–
IVA37	V	B	Pre_ART_	3.1	17	0.2	0.6	41.2	100	50	1	–	0.0	100
			M12_ART_	2.7	16	0.1	0.2	25	100	<1.6	–	–	–	–
IVA38	IV	C	Pre_ART_	3.5	18	0.6	0.7	50	100	6.1	8	0.5	0.4	100
			M12_ART_	2.4	16	0.2	0.4	37.5	100	<1.6	–	–	–	–
IVA40	IV	B	Pre_ART_	3.8	17	0.2	0.6	37.5	94.1	7	2	0.3	0.1	100
			M12_ART_	2.3	10	0	0.1	20	100	<1.6	–	–	–	–
IVA41	V	F1	Pre_ART_	2.3	15	0.1	0.2	26.6	100	4.7	2	0.3	0.1	100
			M12_ART_	<1.6	13	0.1	0.3	30.8	100	2.7	–	–	–	–

*^1^Pre_ART_: before cART start, M12_ART_: 12 months on suppressive cART; ^2^expressed as HIV-1 DNA copies/10^6^ PBMC; ^3^expressed as HIV-1 RNA copies/mL.*

To address the influence of early cART on reservoir complexity, we measured the average pairwise nucleotide differences of the viral population (π) and the uniformity of the haplotype distribution (H_SN_) of viral DNA and RNA from each visit. Hypermutated sequences were excluded from these analyses. All individuals exhibited low π values (<1.5%) despite the Fiebig stage at cART initiation. We observed a minor decline in median proviral π after 1 year of cART (Pre_ART_ = 0.20, IQR: 0.10–0.58 vs. M12_ART_ = 0.10, IQR: 0.20–0.33; *P* = 0.156) (Figure 3B). There was, however, a strong positive correlation between proviral and plasma π values before cART onset (*r* = 0.77; *P* = 0.0157, Supplementary Figure S2), which was not observed at M12_ART_ nor when comparing π values from HIV DNA between visits. The overall median H_SN_ estimated for the HIV-1 proviral population decreased significantly between Pre_ART_ and M12_ART_ visits (0.41, IQR: 0.28–0.56 vs. 0.25, IQR: 0.14–0.30; *P* = 0.019) (Figure 3B). However, no correlation was found when comparing the H_SN_ values calculated from different compartments or visits.

**FIGURE 4:**
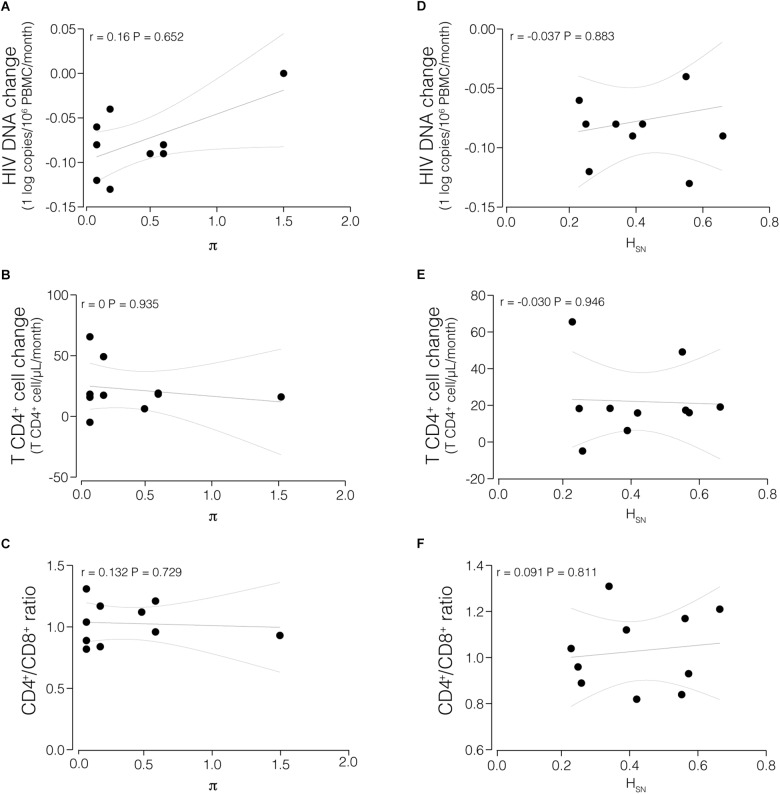
Correlations between proviral HIV-1 diversity indices and immunological and virological measurements. Mean nucleotide diversity (π) of the proviral population at Pre_ART_ was compared with HIV decay in PBMCs **(A)**, T CD4^+^ cell change **(B)**, and CD4^+^/CD8^+^ ratio in M12_ART_ **(C)**. Normalized Shannon entropy (H_SN_) of the proviral population at Pre_ART_ was compared with HIV decay in PBMCs **(D)**, T CD4^+^ cell change **(E)**, and CD4^+^/CD8^+^ ratio in M12_ART_ **(F)**. *P*-values <0.05 were considered statistically significant.

In spite of relatively low π values at Pre_ART_, the values varied among participants (range: 0.1–1.5%). A comparison revealed no significant correlation between π or H_SN_ at Pre_ART_ with immunological and virological parameters, such as rate of HIV DNA decay, T CD4^+^ cell change, or the CD4/CD8 ratio, at M12_ART_ (Figure 4).

## Discussion

A latent HIV-1 reservoir in resting memory CD4+ T cells is recognized as the major barrier to HIV-1 eradication, as its establishment and long-term persistence enables renewed viremia after treatment failure or interruption (Richman et al., 2009). Early cART has been proposed as a means for achieving long-term control of viral replication upon cART interruption, by delaying the viral rebound and potentially inducing a post-treatment controller status (Archin et al., 2012; Avettand-Fènoel et al., 2016). Here, we analyzed ten acutely HIV-infected patients starting cART during the early Fiebig stages, before and 1 year after treatment, to investigate the effect of early therapy on the size and complexity of HIV-1 total DNA in the PBMC reservoir.

Benefits of early cART include a reduction in residual viral replication, a restraint of viral diversity and reservoir development, and accelerated immune restoration (Yerly et al., 2000; Ngo-Giang-Huong et al., 2001; Delwart et al., 2002; Hecht et al., 2006). We observed significant immunological restoration in all subjects 1 year after cART onset during acute HIV infection, with increases of both CD4^+^ T cell counts and the CD4/CD8 ratio (∼1.0), in agreement with previous results (Hoenigl et al., 2016). The rise of the CD4/CD8 ratio was previously linked with reduced levels of HIV DNA in peripheral blood cells (Chun et al., 2002), mainly if cART was established during primary HIV-1 infection (Hocqueloux et al., 2013). Thus, initiating cART during the acute phase of HIV infection offers an opportunity to reduce HIV reservoirs and achieve optimal immune reconstitution. Nevertheless, a recent study (Colby et al., 2018) has demonstrated that a CD4/CD8 ratio <1 is associated with a rapid viral rebound following treatment interruption, in spite of early cART initiation and sustained undetectable viral load along treatment follow-up. The immunological recovery was accompanied here by a reduction in both HIV RNA and DNA levels. The median viral load decay time to undetectable levels was about 3 months after cART start, similar to previous studies (Pilcher et al., 2004; Hoenigl et al., 2016), indicating that a rapid and efficient virologic suppression was achieved in this cohort. The median HIV-1 DNA load of our subjects at baseline visit was ∼3 log copies/10^6^ PBMCs, similar to the one described in the ANRS PRIMO cohort (Schiffer et al., 2004). After 1 year of suppressive cART, HIV-1 DNA levels in PBMCs were reduced to ∼2 log copies/10^6^ PBMCs, reaching similar levels as those of HIV-1-infected patients, who had started intensive and standard cART during primary HIV-1 infection (Hocqueloux et al., 2013; Chéret et al., 2015).

A reduction of the HIV-1 latent reservoir may help HIV-1-infected patients reach at least a transient drug-free remission of their disease (Hocqueloux et al., 2013; Sáez-Cirión et al., 2013). Lower levels of HIV DNA in PBMCs at antiretroviral treatment interruption have been associated with a longer time to treatment resumption (Piketty et al., 2010). Despite the reduction in HIV-1 total DNA in the PBMC reservoir observed in our study, the values were still higher (median 2.18 log copies/10^6^ PBMCs) than in post-treatment controllers (PTC) from the VISCONTI cohort (median 1.71 log copies/10^6^ PBMCs), for whom long-term viral control was maintained after treatment interruption (Sáez-Cirión et al., 2013). The PTC’s median time to plasma viral load becoming undetectable was similar to the one calculated in this study; however, median cART duration was much longer (36.5 months in PTC vs. 12 months in this study). Moreover, a study conducted in Thailand showed that total HIV DNA mean values in PBMCs from early-treated acutely HIV-infected individuals decreased over time achieving very low levels after 3 years of cART (Ananworanich et al., 2016). Thus, with longer follow-up of the patients included in this study, total HIV DNA may reduce even further in the PBMC reservoir.

Here, analysis of HIV *env* sequences from plasma and PBMCs revealed very high population intermixing between the clones obtained before and after cART onset, indicating that the HIV-1 DNA reservoir remained remarkably stable. This result agrees with the findings of Josefsson et al. (2013), whereby viral populations in both pre-therapy plasma and cells isolated after suppressive cART initiated during early/acute infection were nearly monomorphic. The absence of genetic changes indicates that maintenance of the HIV reservoir during suppressive cART, as observed in this study, was the result of homeostatic cell proliferation rather than of ongoing viral replication. This is consistent with the results of other studies analyzing HIV reservoir sequences but from longer time intervals than those reported here (Chomont et al., 2009; Kearney et al., 2014; von Stockenstrom et al., 2015; Brodin et al., 2016; Van Zyl et al., 2017). However, the absence of divergence in the PBMC HIV DNA reservoir does not exclude the possibility that ongoing residual replication could be occurring in sanctuary sites, especially in lymphoid tissue (Lorenzo-Redondo et al., 2016). A more robust sampling scheme, assessing distinct compartments and over a longer time frame should be performed to increase the chances of identifying putatively evolving viruses.

Recent studies have demonstrated that patients, who were treated early during primary HIV-1 infection, had low viral diversity in both plasma or cells isolated after years on therapy (Josefsson et al., 2013; Kearney et al., 2014). Even though diversity values were low at the baseline visit, we detected a slight decline in HIV DNA diversity in PBMCs and a significant decline in proviral population complexity after 12 months on cART. As we found no evidence of ongoing viral replication, the reduction in intrahost proviral complexity following cART may be explained by the clearance of HIV-infected cells, probably long-lived T cells (Palmer et al., 2008), and coincided with a reduction in HIV DNA levels. Interestingly, the diversity indices exhibited by these subjects after 1 year of suppressive cART, were similar to those found in a subgroup of rare individuals capable of naturally controlling HIV-1 replication and maintaining it at low levels (named elite controllers). The diversity indices exhibited by elite controllers are associated with the presence of a putative more efficient mechanism to control HIV-1 replication and disease progression (de Azevedo et al., 2017). A previous study found an association between HIV genetic diversity during the early phase of infection and a faster HIV DNA decline following cART (Wang et al., 2017). In contrast, we did not find any significant correlation between immunological recovery or virologic control achieved after 1 year of suppressive cART and HIV diversity before cART initiation.

We detected three HIV-1 *env* subtypes (B, C, and F1) among the ten patients included in this study. This subtype distribution is in accordance with the molecular epidemiology found in Brazil (Bello et al., 2011). The different HIV-1 clades seem not to have influenced cART outcomes observed in the different subjects. However, it is noteworthy that IVA05 and IVA38, both infected with HIV-1 subtype C strains, presented the highest values of viral diversity and complexity in both PBMC and plasma compartments. This finding agrees with a previous study showing that subtype C displays higher median diversity than subtype B (Rieder et al., 2011). Additionally, the majority of HIV-1 variants were R5-tropic during the entire follow-up period. The only exceptions were two subjects infected with HIV-1 subtype B, who exhibited a low frequency of X4-tropic clones even at the very beginning of HIV-1 infection, in agreement with previous studies (Sheppard et al., 2002; Raymond et al., 2010; Chalmet et al., 2012).

Despite the intensification of offering rapid tests for HIV detection inside and outside the health services, covering key populations at risk, and the ongoing follow-up of cohorts of serodiscordant couples conducted by our institution (INI-FIOCRUZ), the identification of individuals in the early primary infection phases, according to the Fiebig classification, and their follow-up are still hard tasks, as well as, the obtaining of high quality cryopreserved PMBC samples from long term storage to perform the laboratory analyses. Consequently, most of the studies addressing this group, considering the complex strategies for characterizing the early stages of HIV-acute infection, management of early cART starting and the clinical follow-up at specific time points limits the scope, mainly in developing countries where resources to closely following clinical cohorts are more restricted. Thus, the effects of early treatment initiation on the viral evolution, size and distribution of the viral reservoir are still poorly explored and need to be better characterized and, although having a relatively small number of individuals included in our study, we still justified conducting the present analyzes. As of our knowledge, this is the first Brazilian study discussing the viral evolution and reservoir size overtime in a cohort of HIV infected individuals starting cART in the early Fiebig stages of HIV primary infection. Further studies with larger cohorts and longer follow-up periods should be performed to identify better predictive markers for individuals, who might reach the PTC status after cART interruption. Assessment of HIV DNA level and diversity in different T cell subsets and/or compartments would substantially improve our understanding of the effect early cART has in these subjects and would likely add to the growing body of evidence indicating the contribution of each cell population to maintaining the HIV reservoir (Buzon et al., 2014).

In summary, we demonstrate that based on this small cohort and analyses of only the envelope region, 1 year of suppressive cART initiated in the early stages of HIV infection suggested a reduction of the size and complexity of HIV-1 total DNA in the PBMC reservoir, as well as achieve immunological restoration. Immunological recovery and virologic suppression were not associated with proviral diversity before cART onset. The early initiation of cART in HIV-acutely infected individuals in current Brazilian setting may favor strategies to achieve post-treatment control of HIV and, ultimately, a functional cure by restricting the pool of variants and allowing a more focused targeting with therapeutic vaccines or other immune approaches. However, further studies are required to determine the follow-up time necessary to achieve the lowest viral endpoints for clinical management.

## Author Contributions

MG, MM, VV, FC, and BG conceived and designed the study. TL, ED, and FC performed the experiments. AF, SC, and MA participated in patient recruitment. TL, ED, and MG analyzed the data and drafted the manuscript. All authors reviewed and approved the final manuscript.

## Conflict of Interest Statement

The authors declare that the research was conducted in the absence of any commercial or financial relationships that could be construed as a potential conflict of interest.

## Footnotes

1 https://www.hiv.lanl.gov/
