# Supplementary figures and images for: Reduction of HIV-1 Reservoir Size and Diversity After 1 Year of cART Among Brazilian Individuals Starting Treatment During Early Stages of Acute Infection
Source: Front Microbiol. 2019 Feb 11;10:145. doi: 10.3389/fmicb.2019.00145 (PMC6378917; doi:10.3389/fmicb.2019.00145)

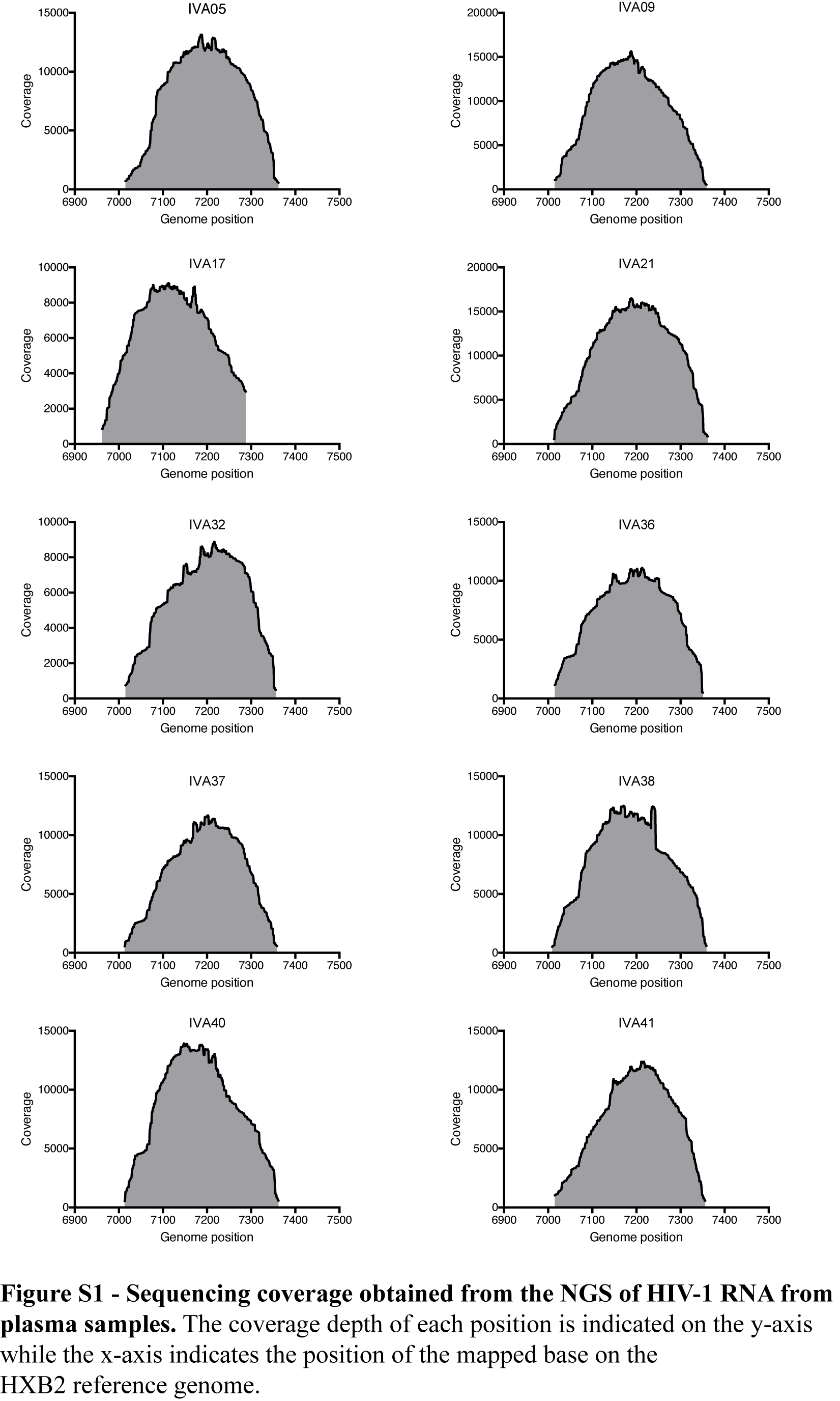

Supplement: Supplementary file 1 [file Image_1.tif]

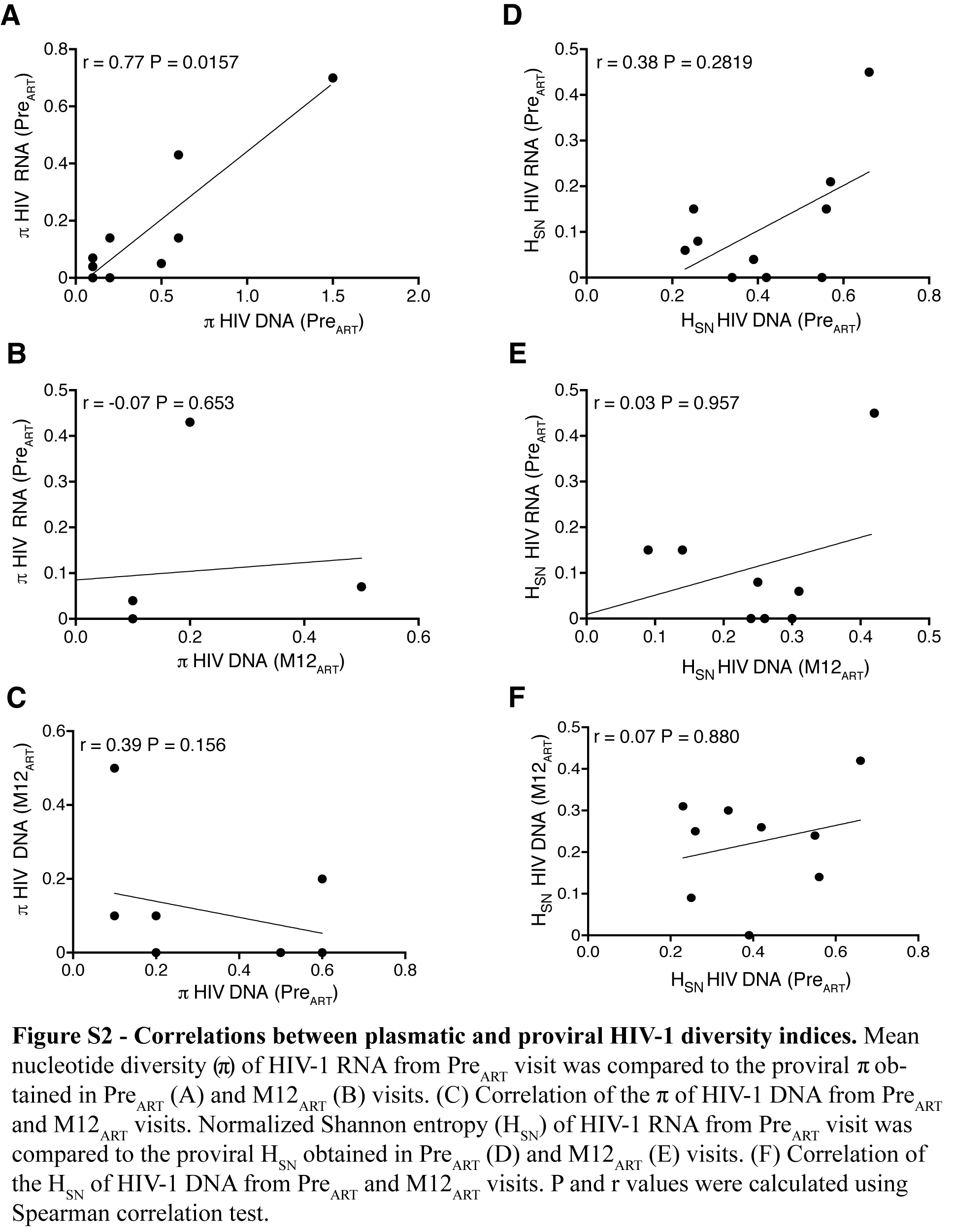

Supplement: Supplementary file 2 [file Image_2.tif]
